# Assessing the Comfort of General Surgery Residents With Various Extremity Splints at Multiple Time Points Before and After a Training Course

**DOI:** 10.7759/cureus.110184

**Published:** 2026-06-03

**Authors:** Jeffrey Doelling, John T Schwartz, Matthew Hatter, Janae Rasmussen, Gary White, Richard Gellman, Eric Huish

**Affiliations:** 1 Orthopedic Surgery, Valley Consortium for Medical Education, Modesto, USA; 2 Surgery, San Joaquin General Hospital, French Camp, USA; 3 Orthopedic Surgery, San Joaquin General Hospital, French Camp, USA; 4 Orthopedics, San Joaquin General Hospital, French Camp, USA

**Keywords:** demonstration course, extremity splinting, musculoskeletal trauma, orthopedic injuries, resident education

## Abstract

Background

In many resident training hospitals, non-orthopedic trainees, such as general surgery residents, participate in early trauma care and may perform initial fracture stabilization. This study evaluated whether a structured splinting curriculum improved general surgery residents’ comfort with identifying and applying common extremity splints.

Methods

Seventeen general surgery residents (seven postgraduate year-1 (PGY-1), one PGY-2, three PGY-3, and two PGY-5) at a community Level II trauma center (San Joaquin General Hospital, California, USA) voluntarily participated in a structured educational session approved by their residency program director. This educational session was part of the general surgery weekly education lecture curriculum and was led by orthopedic residents and an orthopedic technician on June 25, 2025. Resident comfort with identifying and applying splints was measured using a five-point Likert scale at three time points: pre-course, immediately post-course, and six weeks post-course. Changes were analyzed using the Friedman test with Wilcoxon signed-rank post-hoc comparisons and Bonferroni correction.

Results

Fifteen of 17 enrolled residents completed surveys at all three time points (88.2% survey completion). Two residents completed the six-week survey outside of the predefined nine-week cutoff period and were excluded from the final analysis, resulting in a final analytic cohort of 13 residents (76.5% of enrolled participants). Median comfort scores increased from 2 (IQR 1-4) pre-course to 4 (IQR 3-4.25) immediately post-course and remained at 4 (IQR 3-4) at six weeks. Comfort improved significantly across time for all splints (all Friedman p < 0.001). Pairwise comparisons demonstrated significant improvements from pre-course to immediate post-course and from pre-course to six weeks (all p ≤ 0.016), with no significant change between post-course and six weeks.

Conclusions

A brief splinting curriculum significantly improved and sustained general surgery residents’ comfort with splint identification and application.

## Introduction

Extremity fractures are common presentations in emergency departments and trauma settings. In a polytrauma patient, coordinated efforts are required from multidisciplinary care teams, including emergency medicine, general surgery, and orthopedic surgery professionals. In the management of extremity fracture patients, proper initial immobilization with splinting plays a critical role. Specifically, immobilization with a splint contributes to pain control, fracture stabilization, prevention of soft tissue injury, protection of neurovascular structures, and facilitation of safe patient transport prior to definitive orthopedic evaluation and intervention [1]. 

In many resident training hospitals, non-orthopedic trainees (e.g., emergency medicine or trauma surgery residents) frequently perform initial fracture stabilization. Despite the clinical importance of splinting, formal training in splint application is often inconsistent across non-orthopedic residency programs. One study showed that 62% of graduates from a pediatric residency program wanted more experience with applying a temporary splint, and 22% did not apply a splint successfully during training [2]. Another study cited that splints applied by non-orthopedists in an urgent care or emergency room setting were inappropriately applied in 93% of patients [3].Given that musculoskeletal (MSK) complaints comprise approximately 20% of primary care and emergency department visits annually in the United States, adequate splint training modules could be of high value in bridging this knowledge gap for non-orthopedic professionals [4]. 

Furthermore, in splints applied in the emergency room setting, skin and soft tissue complications were found in 40% of patients, and direct injury to the skin and soft tissue, including abrasions, blisters, and ulcerations caused by the splint or elastic bandage, was found in 6% [3]. Inadequate splinting can contribute to patients undergoing additional procedures, enduring unnecessary pain, and medical professionals spending time and resources on healthcare tasks that could have been avoided with proper initial application. Soft tissue complications secondary to inadequate splinting techniques have been found in the literature to be the second most common iatrogenic cause for a plastic surgery referral [5], highlighting the importance of splinting fundamentals when identifying and applying various extremity splints for fracture immobilization. 

Orthopedic literature highlights the importance of proper splint design and application technique. However, educational interventions targeting non-orthopedic trainees remain limited. Simulation-based or hands-on workshops are increasingly used to teach procedural skills in surgical education [6]. At many trauma centers, general surgery residents participate in trauma resuscitations and initial fracture management, often prior to orthopedic consultation. The purpose of this study was to evaluate whether a structured splinting curriculum, led by orthopedic residents and an orthopedic technician, improved general surgery residents’ comfort with (1) identifying appropriate splints and (2) performing splint application at a community Level II trauma center in Northern California (San Joaquin General Hospital). The authors hypothesize that self-reported comfortability scores on the part of the included general surgery residents would increase from pre-course to immediate post-course and remain elevated at the six-week post-course follow-up survey.

The primary objective of the present study was to determine whether a structured splinting curriculum improved general surgery residents’ self-reported comfort with extremity splint identification and application. The secondary objective was to evaluate short-term retention of self-reported comfort at six weeks following the intervention. An exploratory objective was to assess whether changes in comfort differed across individual splint types.

## Materials and methods

The splinting curriculum consisted of a structured, in-person educational session delivered by three senior orthopedic surgery residents (all PGY-3) and an orthopedic surgery technician. The course included a pre-course survey, a didactic review of core splinting principles (“splinting pearls”), live demonstrations, and concluded with hands-on practice of commonly applied upper and lower extremity splints. Didactic content emphasized principles, such as sufficient padding technique, materials selection (e.g., plaster versus orthoglass), limb positioning, and assistant support during immobilization. Demonstrated splints included volar slab, sugar tong, thumb spica, radial gutter, ulnar gutter, posterior long arm, coaptation, short leg with stirrups, Bulky Jones, and long leg with stirrups. Indications for specific splint application based on presenting fracture patterns were also outlined for each individual construct.

Splint course 

A link to the copy of the splint course presentation slides can be found in the Appendix. After the post-course survey, the curriculum also incorporated instruction based on reduction principles, appropriate radiographic assessment (including a minimum two-view radiographic evaluation and specialized views, such as scapular-Y and axillary), and key contraindications (i.e., supracondylar humerus fractures need not be reduced in the emergency setting). Skeletal traction techniques, while not demonstrated and assessed for comfort using the five-point Likert scale, were also reviewed for the distal femur, proximal tibia, and the calcaneus.

Data collection

**Figure 1:**
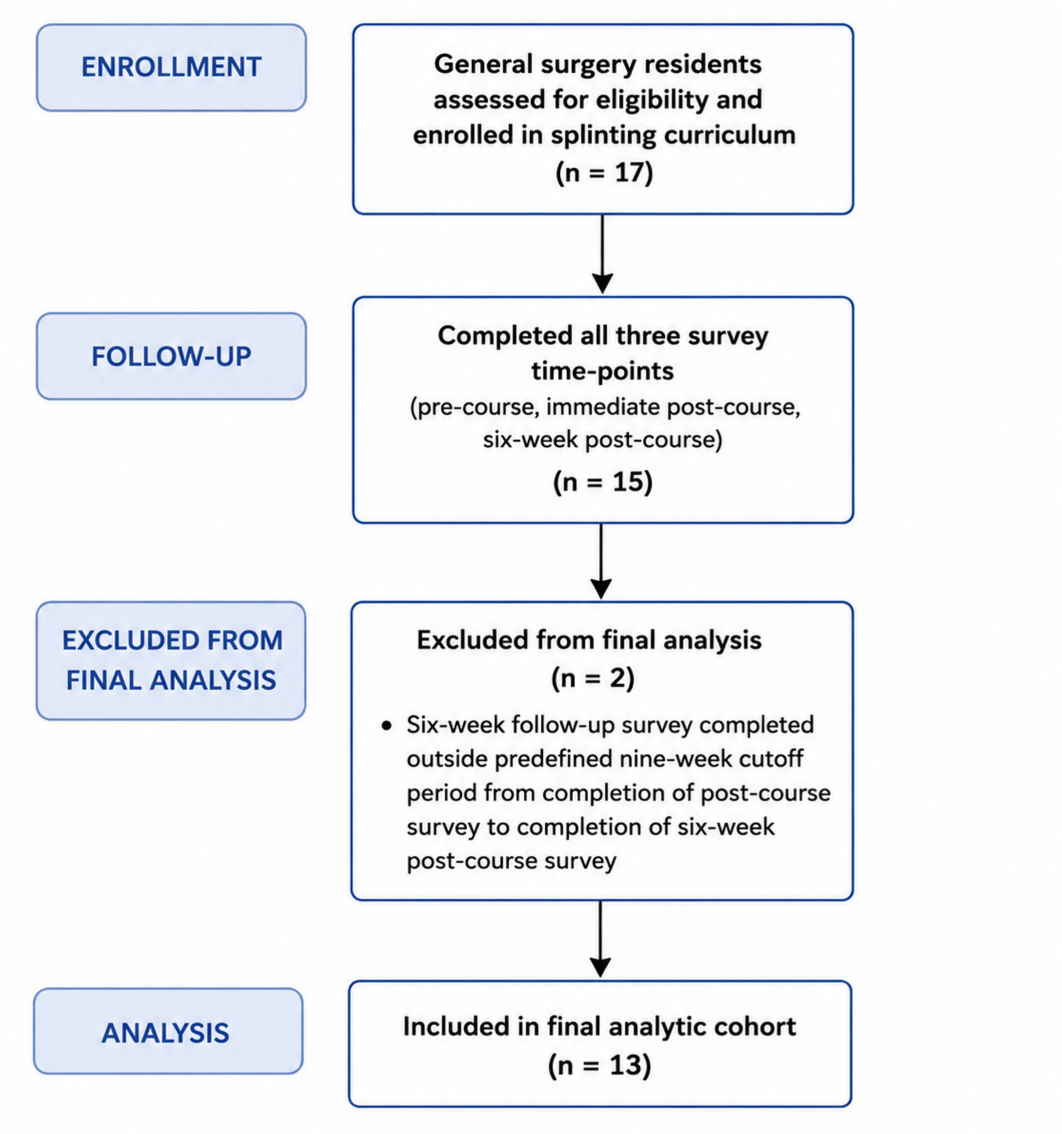
CONSORT flow diagram depicting the final analytical cohort. The predefined cutoff period was nine weeks from the completion of the post-course survey to the completion of the six-week post-course survey.

A total of 17 general surgery residents were initially enrolled in the study cohort. Of these, 15 residents completed surveys at all three designated time points, including the pre-course survey, immediate post-course survey, and six-week post-course survey (see Appendix). The surveys in the present study were developed by the authors solely for the purpose of the present study and were not otherwise distributed to anyone outside of the study participants. Two residents were subsequently excluded from the final analysis because their six-week follow-up surveys were completed outside of the predefined nine-week cutoff period, defined as nine weeks from completion of the immediate post-course survey to completion of the six-week follow-up survey. Therefore, the final analytic cohort consisted of 13 residents. A simplified CONSORT-style flow diagram was added to improve transparency regarding participant inclusion and exclusion throughout the study period and can be found in Figure 1.

**Figure 2:**
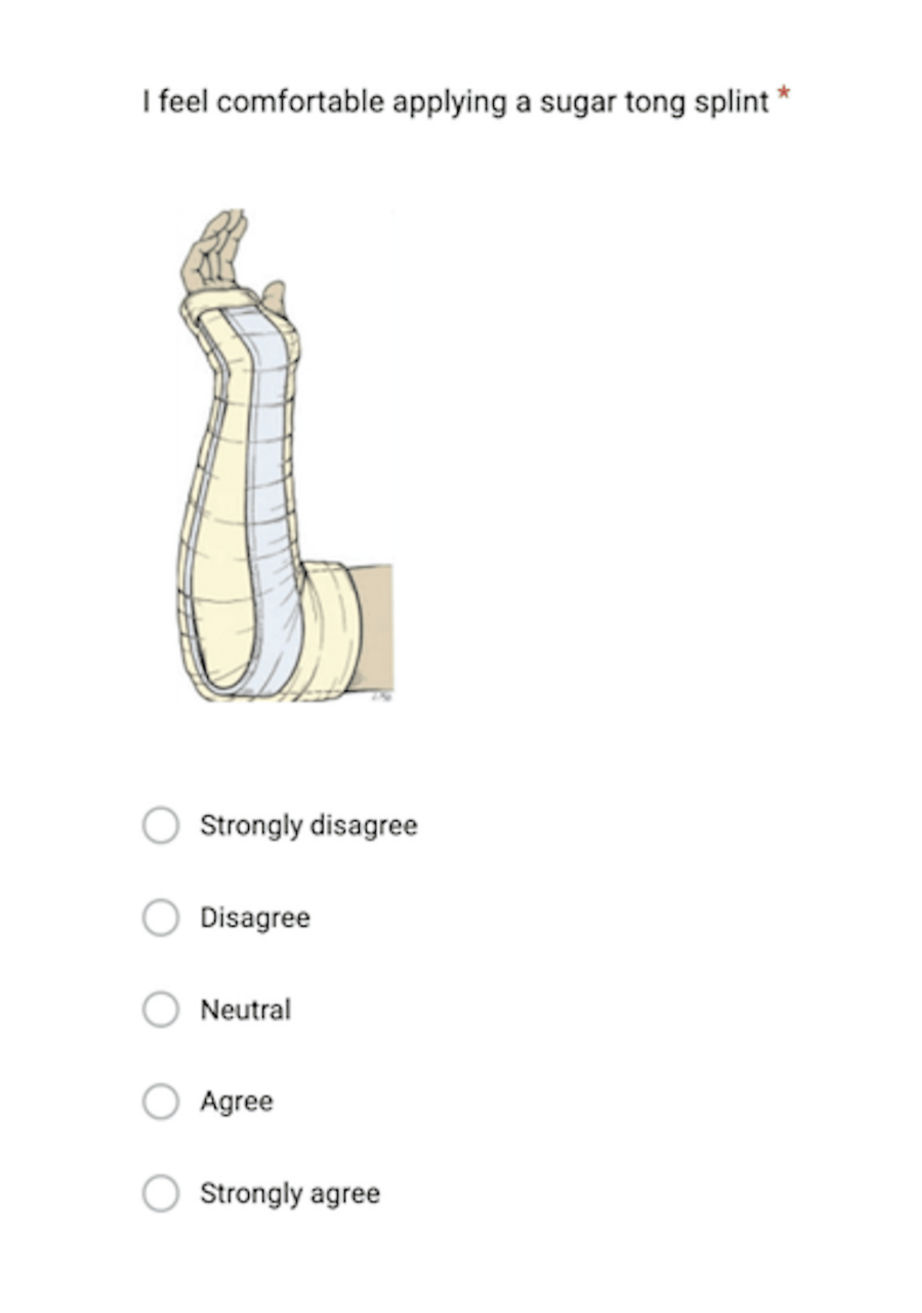
Example question presented to the resident cohort to assess comfortability applying a sugar tong splint.

This study involved measuring comfortability of the same 13 general surgery residents at three time points by means of a five-point Likert scale. The primary outcome measure was the change in the residents' self-reported comfort with splint identification and application following completion of the splinting curriculum. The secondary outcome measure was the retention of self-reported comfort at the six-week follow-up. The pre-course survey, instructional demonstration course, and post-course survey were completed on June 25, 2025. This scale ranged from strongly disagree, to disagree, to neutral, to agree, to strongly agree when asked how comfortable the resident was with identifying and performing the correct splint. Because these responses represented ordinal, non-normally distributed data with greater than two related samples from each resident (one from each time point), the Friedman test was employed to provide insight on if comfort level changed across the three time points (pre-training course, immediately post-training course, and six weeks post-training course). Data points were included if the six-week post-training course survey was completed within nine weeks of the training course. On average, the six-week post-training course was completed 8.62 weeks (60.34 days) after the training course. A representative image of the comfortability assessment can be found in Figure 2.

Statistical analysis

Pairwise comparisons with the Wilcoxon signed-rank tests with Bonferroni correction were then completed to compare the following: 1) pre-training course versus immediate post, 2) pre-training course vs. six-week post, and 3) immediate post-training course vs. six-week post. The adjusted p-value after Bonferroni correction was α = 0.05/3 = 0.017. 

The comfortability response scale ordinal data were assigned numeric values as follows: strongly disagree = 1, disagree = 2, neutral = 3, agree = 4, strongly agree = 5. A separate Friedman test with subsequent Wilcoxon signed-rank test for post-hoc analysis was run for each of the splint types. The software JASP was used to run all of the statistical tests (JASP Team, 2025, version 0.19.3). Changes in comfort level for each splint type across the three time points (pre-course, immediate post-course, and six-week follow-up) were analyzed using a Friedman test for repeated ordinal measures.

## Results

A total of 17 general surgery residents at a community Level II trauma center in Northern California were enrolled to participate in the study. Two residents provided incomplete data for the three surveys and were excluded. The remaining 15 residents completed surveys at all three time points (88.2% follow-up). However, two of these 15 residents completed the six-week survey outside of the nine-week cut-off point and were thus excluded (response rate 13/17; 76.47%). Seven of these residents were postgraduate year-1 (PGY-1), one was a PGY-2, three were PGY-3, and two were PGY-5. 

The median comfort level increased from 2 (IQR 1-4) prior to the training course to 4 (IQR 3-4.25) after the training course and remained at 4 (IQR 3-4) six weeks after the training course (Table 1). 

**Table 1:** Resident comfort level with splint application over time.

Time point	Median (IQR)
Pre-training course	2 (1-4)
Immediately post-training course	4 (3-4.25)
Six-week post-training course	4 (3-4)

Median comfort levels increased significantly across time for all splint types evaluated (all Friedman p < 0.001) (Table 2).

**Table 2:** Comparison of comfort levels across time points (N = 13). * Denotes statistical significance

Splint type	Friedman p	Pre vs. post	Pre vs. six weeks	Post vs. six weeks
Volar	0.000014	0.002*	0.002*	0.046
Thumb spica	0.000094	0.003*	0.004*	0.180
Sugar tong	0.000455	0.004*	0.006*	0.317
Posterior long arm	0.0005	0.004*	0.004*	0.739
Coaptation	0.00054	0.004*	0.016*	0.102
Short leg	0.000086	0.002*	0.004*	0.317
Long leg	0.000683	0.007*	0.004*	0.317
Selecting the correct splint	0.00013	0.002*	0.004*	0.527

Separate Friedman tests were performed independently for each splint type to evaluate changes in resident self-reported comfortability across the three study time points (pre-course, immediate post-course, and six-week post-course). Pairwise comparisons with Bonferroni correction (adjusted significance threshold α = 0.017) demonstrated significant improvement from pre-course to immediate post-course assessment for all splints, including volar, thumb spica, sugar tong, posterior long arm, coaptation, short leg with stirrups, and long leg with stirrups splints (all p-values ranged from 0.002 to 0.007) (Table 2). Improvements from pre-course to six-week follow-up remained statistically significant for all splints (all p-values ranged from 0.002 to 0.016), indicating sustained retention of comfort with splint application (Table 2). There were no statistically significant differences between immediate post-course and six-week assessments for any splint type (all p-values > 0.017), suggesting no measurable decline in self-reported comfort over time (Table 2).

In the interval from the immediate post-training course survey to the six-week post-training course survey, seven residents spent zero weeks on the trauma service and did not perform splinting. One resident spent four-to-five-day shifts on the trauma service and performed splinting. One resident spent two weeks on the trauma service but did not perform splinting. Two residents spent three weeks on the trauma service but did not perform splinting. One resident spent four weeks on the trauma service and performed splinting. One resident spent an unspecified number of weeks on the trauma service and performed splinting. Overall, three residents in the final analytic cohort performed splinting during the interval between the immediate post-course survey and the six-week follow-up survey.

## Discussion

The primary finding of the present study was that a brief, structured splinting curriculum, complete with live demonstrations and hands-on practice, significantly improved general surgery residents’ self-reported comfort with splint identification and application. Notably, comfortability improvements were seen across all splint types evaluated, including volar slab, sugar tong, thumb spica, radial gutter, ulnar gutter, posterior long arm, coaptation, short leg with stirrups, and long leg with stirrups. Increased comfort persisted at the six-week follow-up, suggesting short-term retention of knowledge and skills gained at the splinting workshop demonstration course. Importantly, the Likert-based comfortability assessments used in the present study should be interpreted as a measure of self-reported confidence rather than objective procedural competence or technical proficiency. These findings support the concept that procedural confidence with splint identification and application can improve with short, targeted teaching sessions.

A number of different studies have established that training through simulation markedly improves knowledge acquisition and technical competency development within surgical education [7,8]. McGaghie and colleagues completed a meta-analysis that found large, statistically significant improvements in procedural and technical skills when simulation-based medical education practices were used [9]. The splint workshop in the present study combined didactic review of principles and pitfalls, demonstrations by experienced instructors, and hands-on practice in a short, hour-and-a-half-long training course. However, unlike prior simulation-based procedural studies incorporating objective performance assessments, the present study did not directly evaluate splint quality, technical accuracy, or procedural competency. Therefore, conclusions should be limited to improvements in resident-reported comfort and confidence rather than confirmed improvements in technical skill acquisition.

Because splinting is commonly performed by providers from multiple speciality backgrounds, cross-speciality training programs designed by the orthopedic department may result in improved patient care during trauma activations, reduced delays in immobilization, and improved communication across multidisciplinary care teams. Notably, most general surgery residents included in the present study did not perform splinting in the interval from the post-course survey to the six-week post-course survey. Comfort scores in choosing and applying the correct splints nonetheless remained elevated. This suggests that the course may have provided durable, foundational concept understanding that persisted even without immediate and repetitive clinical reinforcement. While sustained confidence may represent improved familiarity with splint selection and application principles, this cannot be assumed to correlate directly with sustained procedural competency in the absence of objective technical assessment. In addition, retention outcomes cannot be attributed solely to the educational intervention, as residents had heterogeneous exposure to trauma service and splinting opportunities during the follow-up interval. Exposure ranged from no trauma service participation to up to four weeks of trauma service experience, with varying levels of splint application performed clinically. This variability represents a potential confounding factor that may have independently influenced comfort retention estimates at six weeks. This result also highlights a potential role for periodic refresher sessions to maintain procedural competency in training non-orthopedists in splinting techniques.

Given that the splint training demonstration course required minimal time, limited equipment, and a small number of instructors, it would be easily reproducible at other institutions due to its simplicity. Orthopedic senior residents and technicians are well-positioned to teach these skills to other medical professionals. While high-acuity trauma management is a cornerstone of general surgery residency curricula across the country, technical aspects of fracture immobilization are more commonly taught in orthopedic surgery resident education. Incorporating splinting education early on in general surgery residency might improve trainee confidence in the ability to manage MSK injuries, reduce reliance on orthopedic consultation for basic immobilization, and improve patient throughput in emergency and trauma settings. In a 2011 retrospective cross-sectional study of a stratified random sampling of 35,849 patient visits to 364 non-Federal U.S. hospital EDs in 2006 by Horwitz and colleagues, a minority of hospitals consistently achieved recommended wait times for all ED patients, and fewer than half of hospitals consistently admitted their ED patients within six hours [10]. However, the generalizability of the present findings should be interpreted cautiously given the single-institution design, small analytic cohort, and the specific educational environment of a community Level II trauma center. Institutional variability in orthopedic exposure, trauma volume, access to orthopedic support staff, and baseline splinting education across residency programs may influence both implementation feasibility and educational outcomes. In addition, academic tertiary referral centers, smaller community hospitals, and resource-limited healthcare systems may differ substantially in their ability to provide dedicated faculty time, orthopedic technician involvement, simulation materials, and protected educational sessions for procedural splinting curricula.

This study is not without limitations. First and foremost, the small sample size of 13 general surgery residents limits statistical power and generalizability. Furthermore, this is a single-institution study at a community Level II trauma center. The study also did not control for postgraduate year level, prior splinting experience, or heterogeneous trauma service exposure during follow-up, all of which may have influenced resident comfort retention and learning outcomes. In addition, outcomes relied on self-reported comfortability rather than objective skill assessment. As a result, the included general surgery residents may have experienced response bias or social desirability bias when reporting confidence. Importantly, increased self-reported comfort does not necessarily equate to improved technical performance or procedural accuracy. Residents may feel more confident despite persistent deficiencies in splint construction technique or immobilization quality. Lastly, the study did not assess the accuracy of splint constructions, the quality of immobilization, or patient outcomes. Objective assessment was not performed because of limited faculty availability, time constraints during the educational session, and lack of a validated institutional splint assessment tool at the time of study initiation. Future iterations of this curriculum should incorporate structured objective evaluation methods, such as checklist-based scoring systems, OSCE (Objective Structured Clinical Examination)-style assessments, blinded faculty grading of splint quality, or standardized competency rubrics evaluating padding, mold quality, fracture immobilization, and overall splint appropriateness. Future research should emphasize evaluating splint quality objectively using standardized checklist assessments after training courses are completed. The curriculum could also be expanded to include other medical specialties that often provide care for musculoskeletal injury patients, including but not limited to emergency medicine residents, family medicine residents, physician assistants, and medical students. Studying longer-term retention (six to 12 months), with standardized, objective evaluations of splint quality, could also provide insights as to whether splint training leads to improved clinical outcomes and/or reduced splint complications.

## Conclusions

The results of the present study suggest that brief, focused educational interventions can improve self-reported confidence in splint identification and application among non-orthopedic surgical trainees. However, these findings reflect resident-reported comfort rather than objective procedural competency, and retention outcomes may have been influenced by variable clinical exposure to splinting during follow-up. Future multi-institutional studies incorporating objective competency assessments are necessary. In addition, future research incorporating needs assessment surveys of non-orthopedic residency program directors may better define the perceived educational and clinical value of formal splinting curricula for non-orthopedic trainees.

## Appendices

The splint course presentation can be accessed at https://docs.google.com/presentation/d/1iy-IbiN0B8lENt5A8cbFS5-23HzNZcB3IY8G-a8aU8I/edit?usp=sharing.

The pre-course survey can be accessed at https://docs.google.com/forms/d/1Psbb9TyIX1boGRnvaXjC8XYgzsKkIuwV3Oz1xf2pTAs/edit.

The immediate post-course survey can be accessed at https://docs.google.com/forms/d/1ibjnOHguszSbwV5MfjZYlmUTF2O-XK-d0oRYn9_OS14/edit.

The six-week post-course survey can be accessed at https://docs.google.com/forms/d/1grQ0xU6YVe0PeUpF04vNrZo8dAa1VdnzV8eCvgcVaJM/edit.

The surveys in the present study (pre-course survey, immediate post-course survey, and six-week post-course survey) were developed by the authors solely for the purpose of the present study, and were not otherwise distributed to anyone outside of the study participants.
